# Dynamic modeling and optimal control of Mpox among MSM in a central Chinese city

**DOI:** 10.1186/s12889-026-28925-6

**Published:** 2026-08-15

**Authors:** Dan Wang, Si Wu, Siyu Zhang, Han Ni, Yuxi He, Liuyi Fu, Jin Zhao, Yuan Lv, Wenting Zha

**Affiliations:** 1 Hunan Key Laboratory of Molecular Epidemiology, Hunan Normal University Health Science Center, Changsha, Hunan Province 410013 China; 2 Infectious Disease Early Warning and Decision Center, Hunan Normal University Health Science Center, Changsha, Hunan Province China; 3 Hunan Provincial Center for Disease Control and Prevention, Changsha, Hunan Province China; 4 Changsha Center for Disease Control and Prevention, Changsha, Hunan Province China

**Keywords:** Mpox virus, Dynamic models, Optimal control, Interventions, Epidemiology

## Abstract

**Background:**

Since 2022, the mpox outbreak has become a global public health threat, with transmission mainly among men who have sex with men (MSM). Mpox is the only infectious disease other than Ebola to have been declared a PHEIC twice. Following its importation and local spread in China, developing prevention strategies is essential. This study aims to formulate the optimal control strategy for mpox within MSM population in urban China.

**Methods:**

We developed a compartmental transmission dynamics model for mpox among MSM in a Chinese city. The framework extends SEIR by including asymptomatic infections (*A*) and symptomatic but undiagnosed infections (*B*) alongside *S*, *E*, *I*, *R*, with transmission arising from *A*, *I*, *B*. We then formulated an optimal control problem with 4 interventions: MSM community based prevention and control (*u*_1_), Early detection/Early treatment/Isolation (*u*_2_), Increasing medical consultation rate (*u*_3_), and Vaccination (*u*_4_), and evaluated 15 schemes over 100 days. In addition, we set several constant control combinations and compared their costs and effects with optimal control.

**Results:**

In the no-control scenario simulation, cumulative infections reached 6331 by day 100. Across 15 optimal control schemes ranging from single to quadruple controls, implementing any scheme reduced total infections effectively. With a single control measure, *u*_1_ achieved the best performance and averted 6238 infections. For dual control measures, *u*_1_ + *u*_4_ showed the strongest effect and averted 6250 infections. For triple control measures, *u*_1_ + *u*_3_ + *u*_4_ averted 6256 infections. Quadruple control measures provided the most effective overall strategy and averted 6258 infections. The quadruple constant control settings C1–C5 averted 5980, 6202, 6154, 6165 and 6181 infections, respectively. Under quadruple controls, the accumulated control-only cost was 1209.57 for optimal control. The costs of constant control settings C1–C5 were 1260.00, 2845.00, 3600.00, 3015.00, and 4575.00, respectively.

**Conclusions:**

The no-control simulation suggests that mpox transmission among MSM can produce a large number of infections. The optimal control measures can effectively control the epidemic. MSM community based prevention and control (*u*_1_) is the best single control measure. Quadruple control measures avert the largest number of infections overall. Compared with constant control measures, optimal control achieves higher effectiveness at comparable cost.

**Trial registration:**

Clinical trial number: not applicable.

**Supplementary Information:**

The online version contains supplementary material available at https://doi.org/10.1186/s12889-026-28925-6.

## Background

Mpox is a zoonotic infectious disease caused by the monkeypox virus (MPXV) [1]. Clinically, mpox presents with symptoms including rash, fever, and lymphadenopathy [2]. The latent period generally ranges from 5 to 21 days [3]. Mpox used to be prevalent only in Africa. However, since May 2022, outbreaks have been reported globally, posing a significant threat to global public health [4]. The World Health Organization (WHO) declared the mpox outbreak a Public Health Emergency of International Concern (PHEIC) in July 2022 and August 2024, making it the only infectious disease besides Ebola to have been designated a PHEIC twice [5]. The 2022 PHEIC was mainly associated with the global outbreak of MPXV clade IIb, whereas the 2024 PHEIC was related to the upsurge of clade I/clade Ib mpox in the Democratic Republic of the Congo and other African countries [6, 7]. By January 2026, the WHO had reported a total of 179,612 confirmed cases and 483 deaths worldwide [8].

The 2022 mpox outbreak is associated with changes in both viral biology and human behaviors [9]. Contributing factors include waning smallpox immunity, relaxation of COVID-19 preventive measures, resumption of international travel, changes in sexual behaviors, and occurrence of large gatherings [10, 11]. Available data indicate that the primary transmission route of the 2022 mpox outbreak is human-to-human, with the epidemic predominantly concentrated within the men who have sex with men (MSM) community, accounting for over 98% of male cases and more than 95% within MSM populations worldwide [10]. In China, the first indigenous mpox case was reported on June 2, 2023 [12]. On September 20, 2023, China incorporated mpox into category B infectious diseases. By November 30, 2023, China had reported a total of 1611 confirmed cases of mpox, all of which were attributed to MPXV clade IIb; 99.3% of the cases occurred in males, and 94.7% were among men who have sex with men (MSM) [13]. Therefore, MSM are a priority population in mpox prevention and control. To reduce mpox transmission, China implemented prevention and control measures, including early detection, early reporting, early isolation and early treatment, surveillance through multiple channels, health education and comprehensive interventions for key populations. For MSM populations, these measures mainly included peer education, online health education, promotion of safer sexual behaviors, promotion of testing and outreach services, among others [14]. Acquired immunity may reduce the susceptible population and lower the effective reproduction number, thereby slowing epidemic growth and contributing to outbreak decline [15]. To date, mpox vaccine development has been actively supported in the Chinese mainland, and no mpox vaccine has yet received marketing approval [16].

Mathematical modeling plays an important role in understanding the transmission of infectious diseases. Specifically, the infectious disease dynamics models provide valuable frameworks for analyzing the spread of mpox and evaluating the effectiveness of interventions. Since the 2022 mpox outbreak, several studies worldwide have developed models to estimate basic reproduction numbers and assess intervention strategies [17]. Yuan et al. [18] constructed an SEIR-based compartmental model for mpox transmission in a hypothetical metropolitan population, distinguishing high- and low-risk groups to assess transmission risks and public health control strategies. Endo et al. [19] used a transmission model fitted to empirical sexual partnership data and showed that heavy-tailed sexual contact networks could support sustained mpox transmission among MSM. Xiridou et al. [20] developed a transmission dynamics model stratifying MSM population by sexual activity levels and accounting for behavioral adaptations. Gregory Gan et al. [21] used an SEIR model to simulate outbreaks under various sexual contact networks and assess vaccination impact within MSM communities.

Combining infectious disease dynamics models with optimal control theory provides an approach to find the measures that achieve effective infection control with minimal cost. Many researchers have applied it to investigate the optimal intervention strategies. Ahmad et al. [22] formulated an optimal control system incorporating three mpox interventions, showing that vaccination alone after the latent period has limited impact, whereas combined strategies involving pre-incubation vaccination and isolation are more effective. Yapiskan et al. [23] proposed a novel mpox transmission model identifying optimal control strategies that significantly reduce infections through combinations of two adaptive controls. Samreen et al. [24] concluded that implementing all four control measures simultaneously maximizes reduction of community-level infections.

Given limited public health resources in many low and middle income countries, practical models that incorporate local epidemic characteristics can help prioritize feasible combinations of control measures for vulnerable populations and support strategy formulation. Against this background, this study aims to develop a practical model tailored to China’s epidemic situation. In medium and large cities in China, mpox outbreaks are commonly initiated by imported cases and primarily lead to local transmission within MSM populations. In addition, we noted that asymptomatic infections and undiagnosed symptomatic individuals may play important roles in mpox transmission. Therefore, this study develops a mpox transmission dynamics model that explicitly accounts for asymptomatic infections and undiagnosed symptomatic individuals, incorporating estimated MSM population data from a central Chinese city. Furthermore, to design effective control strategies, we extend the model to include 4 interventions (control measures) and apply optimal control theory to evaluate their effectiveness, thereby determining the optimal prevention and control scheme.

## Methods

### Transmission model without optimal control

The transmission process of mpox consists of several stages: susceptible, exposed, infected (including symptomatic and asymptomatic infections), and recovered. Most mpox cases are found within the MSM population, with primary transmission routes being sexual contact and close physical contact. Currently, the majority of confirmed mpox cases are diagnosed through active healthcare seeking. However, some infected individuals exhibit symptoms but remain undiagnosed and untreated, potentially causing hidden transmission within the MSM community. Therefore, this model differentiates symptomatic infections into two compartments: diagnosed symptomatic individuals and undiagnosed symptomatic individuals.

The transmission dynamics model is constructed focusing on the MSM population in a central city. Building upon the classic SEIR framework, the model incorporates both asymptomatic infected individuals and undiagnosed symptomatic individuals to better represent the characteristics of mpox transmission. The model consists of six compartments: susceptible (*S*), exposed (*E*), asymptomatic (*A*), symptomatic and diagnosed (*I*), symptomatic but undiagnosed (*B*), and removed (*R*).

**Fig. 1:**
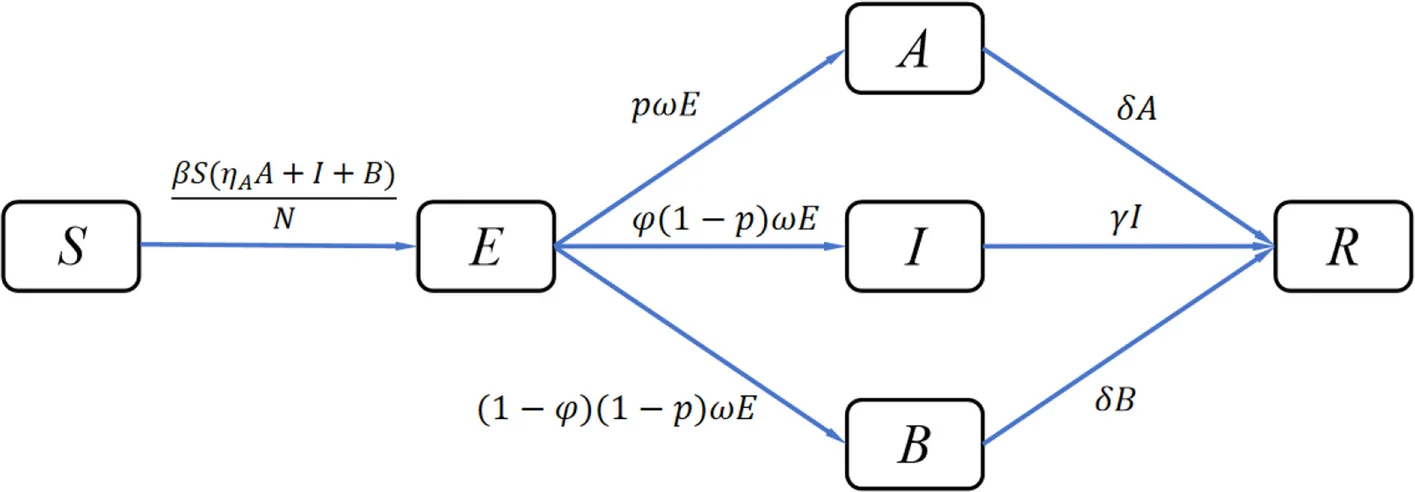
Flow diagram of the mpox transmission in MSM

Based on virological, epidemiological, and clinical characteristics of mpox, a transmission dynamics model (1) without control measures is established under the assumption that the entire MSM population in the city is susceptible. The flow diagram of the model is shown in Fig. 1. The model is based on the following assumptions and facts:

1. Population birth and natural death rates are not considered, the total MSM population denoted by *N* = *S* + *E* + *A* + *I* + *B* + *R*.
2. Disease-induced mortality and infectiousness during the latent period are not considered.
3. Asymptomatic infected individuals (*A*), symptomatic diagnosed individuals (*I*), and symptomatic undiagnosed individuals (*B*) are all infectious. Susceptible individuals become infected upon contact with any infectious individual.
4. The proportion of asymptomatic infections among all infections is denoted by *p*. The baseline transmission rate, corresponding to symptomatic, is denoted by *β*. The transmission rate of asymptomatic infected individuals (*A*) is given by *η*_*A*_*β*.
5. The latent period of mpox is *1/**ω*. After the latent period, individuals transition to asymptomatic infected (*A*), symptomatic diagnosed (*I*), or symptomatic undiagnosed (*B*) compartments with proportions $p\omega E, \varphi (1-p)\omega E and (1-\varphi )(1-p)\omega E$, respectively.
6. Symptomatic infected individuals are divided into diagnosed and undiagnosed groups; the proportion of diagnosed individuals is *φ*.
7. The time to diagnosis for symptomatic individuals is *1/γ*. Once diagnosed, individuals are immediately removed from the infectious population.
8. The recovery rates for undiagnosed symptomatic individuals and asymptomatic infected individuals are denoted by *δ*.

The system of ordinary differential equations for Model (1) is as follows:

1 $$\left\{\begin{array}{c}\frac{dS}{dt}=-\frac{\beta S({\eta}_{A}A+I+B)}{N}\\ \frac{dE}{dt}=\frac{\beta S({\eta}_{A}A+I+B)}{N}-\omega E\\ \frac{dA}{dt}=p\omega E-\delta A\\ \frac{dI}{dt}=\varphi (1-p)\omega E-\gamma I\\ \frac{dB}{dt}=(1-\varphi )\left(1-p\right)\omega E-\delta B\\ \frac{dR}{dt}=\gamma I+\delta (A+B)\end{array}\right.$$

The definitions and ranges of the model parameters are listed in Table 1.

**Table 1:** Parameters of model with values

Parameter	Description	Value	Source
*β*	Transmission rate	0.1232	Estimated
*ω*	Progression from exposed to infected class	0.2	[39]
*p*	Proportion of asymptomatic infections	0.135	[41]
*φ*	Proportion of diagnosed symptomatic cases	0.35	[40]
*γ*	Removal rate of diagnosed patients	0.15	[39]
*δ*	Recovery rate of undiagnosed symptomatic and asymptomatic infected individuals	0.05	[42]
${\eta}_{A}$	Relative infectiousness of asymptomatic infections	0.5	[43]
*N*	MSM population size	81,224	Estimated

### Positivity and boundedness of solutions

For Model (1), let the initial conditions satisfy $S\left({0}\right)\ge {0}$, $E\left({0}\right)\ge {0}$, $A\left({0}\right)\ge {0}$, $I\left({0}\right)\ge {0}$, $B\left({0}\right)\ge {0}$, and $R\left({0}\right)\ge {0}$, with $N\left({0}\right)>{0}$. Since the right-hand side of Model (1) is locally Lipschitz continuous in the feasible region, the solution exists and is unique. To verify positivity, we evaluate the system on the boundary of each compartment:

$${\left.\frac{dS}{dt}\right|}_{S=0}=0, {\left.\frac{dE}{dt}\right|}_{E=0}=\frac{\beta S\left({\eta}_{A}A+I+B\right)}{N}\ge 0,$$

$${\left.\frac{dA}{dt}\right|}_{A=0}=p\omega E\ge 0, {\left.\frac{dI}{dt}\right|}_{I=0}=\varphi \left(1-p\right)\omega E\ge 0,$$

$$\begin{aligned} {\left.\frac{dB}{dt}\right|}_{B=0} &=\left(1-\varphi \right)\left(1-p\right)\omega E\ge 0, {\left.\frac{dR}{dt}\right|}_{R=0}\\ &=\delta A+\gamma I+\delta B\ge 0. \end{aligned}$$

Therefore, all state variables remain non-negative for *t≥ 0*. Furthermore, by summing all equations in Model (1), we obtain

$$\frac{dN}{dt}=\frac{d}{dt}\left(S+E+A+I+B+R\right)=0.$$

Thus, $N\left(t\right)=N\left(0\right)$ for all *t≥ 0*, and each compartment is bounded above by $N\left(0\right)$. Hence, the feasible region

$$\begin{aligned} \Omega &= \{\left(S, E, A, I, B, R\right):S, E, A, I, B, R\ge 0,\\ &\ \quad S+E+A+I+B+R=N\left(0\right)\}. \end{aligned}$$

is positively invariant. This indicates that Model (1) is epidemiologically well posed.

### Basic reproduction number

The basic reproduction number (*R*_*0*_) is widely used to evaluate the transmissibility of infectious diseases. It quantifies the average number of secondary cases generated by a single infectious individual during their infectious period in a fully susceptible population [25]. In this study, the next-generation matrix method [26] is employed to calculate *R*_*0*._ The compartments in the model are categorized into two groups: uninfected compartments (*S, R*) and infected compartments (*E, A, I, B*). According to the next-generation matrix approach, taking the partial derivatives of *F* and *V* with respect to the infected compartments *E*, *A*, *I* and *B*, the Jacobian matrices at the disease-free equilibrium are obtained as:

$$F\mathrm{=}\left[\begin{array}{cccc}{0}& {\eta}_{\mathrm{A}}\beta & \beta & \beta \\ {0}& {0}& {0}& {0}\\ {0}& {0}& {0}& {0}\\ {0}& {0}& {0}& {0}\end{array}\right]$$

and

$$V=\left[\begin{array}{cccc}\omega & 0& 0& 0\\ -\omega p& \delta & 0& 0\\ -\omega \varphi (1-p)& 0& \gamma & 0\\ -\omega (1-p)(1-\varphi )& 0& 0& \delta \end{array}\right]$$

Since the basic reproduction number *R*_*0*_ is the spectral radius of the next-generation matrix ${FV}^{-1}$, we have:

2 $${R}_{0}\mathrm{=}\rho \left(F{V}^{-1}\right)= \mathrm{ } \beta \mathrm{[}\frac{p{\eta}_{A}}{\delta }\mathrm{+}\frac{\mathrm{(1-}p\mathrm{)}\varphi }{\gamma }\mathrm{+}\frac{\mathrm{(1-}p\mathrm{)(1-}\varphi \mathrm{)}}{\delta }\mathrm{]}$$

### Threshold interpretation of the basic reproduction number

Model (1) has a continuum of disease-free equilibria, given by ${\varepsilon}_{0}=\{\left(S,\mathrm{0,0},\mathrm{0,0},R\right):S+R=N\}$. For the calculation of the basic reproduction number, the next-generation matrix is evaluated at the fully susceptible disease-free state ${\varepsilon}_{0}\mathrm{=}\left(N\mathrm{,0,0,0,0,0}\right)$. Therefore, the basic reproduction number *R*_*0*_ represents the expected number of secondary infections generated by a typical newly infected individual introduced into an otherwise fully susceptible MSM population.

As a threshold quantity in compartmental infectious disease models, *R*_*0*_ characterizes the local invasion potential of the infection. According to the standard next-generation matrix framework, when *R*_*0*_ < 1, the infected subsystem is locally asymptotically stable at the fully susceptible disease-free state ${\varepsilon}_{0}=\left(N,\mathrm{0,0},\mathrm{0,0},0\right)$ and small introductions of infection are expected to die out; when *R*_*0*_ > 1, infection can invade the fully susceptible population during the early phase of transmission.

The analytical expression of *R*_*0*_ further indicates that transmission in Model (1) is jointly driven by three infectious compartments: asymptomatic infected individuals (*A*), symptomatic and diagnosed individuals (*I*), and symptomatic but undiagnosed individuals (*B*). Accordingly, parameters governing the transmission coefficient *β*, the proportion of asymptomatic infections *p*, the relative infectiousness of asymptomatic infections *η*_*A*_, the proportion of diagnosed symptomatic cases *φ*, the removal rate of diagnosed individuals *γ*, and the recovery rate of asymptomatic infected individuals and symptomatic but undiagnosed individuals *δ* affect the magnitude of *R*_*0*_.

### Sensitivity analysis

To investigate the influence of different parameters on the spread of mpox, the sensitivity index was used to analyze the sensitivity of the basic reproduction number (*R*_*0*_) to parameters. The sensitivity index is defined as:

$${\xi}_{x}^{f}=\frac{x}{f}\times \frac{\partial f}{\partial x}$$

Based on the analytical expression of the basic reproduction number (${R}_{0}$), and excluding uncontrollable variables, we examined the sensitivity of ${R}_{0}$ to the transmission coefficient *β*, the proportion of asymptomatic infections *p*,the proportion of diagnosed symptomatic cases *φ*, the removal rate of diagnosed individuals *γ*, and the recovery rate of asymptomatic infected individuals and symptomatic but undiagnosed individuals *δ*, the relative infectiousness parameter ${\eta}_{A}$. The partial derivatives of ${R}_{0}$ with respect to these parameters are:

$$\begin{aligned} &\frac{\partial {R}_{0}}{\partial \beta }=[\frac{p{\eta}_{A}}{\delta }+\frac{(1-p)\varphi }{\gamma }+\frac{(1-p)(1-\varphi )}{\delta }]>0,\\ &\frac{\partial {R}_{0}}{\partial p}=-\frac{\beta \left[\delta \varphi +\gamma \left(1-\varphi -{\eta}_{A}\right)\right]}{\gamma \delta },\\ &\frac{\partial {R}_{0}}{\partial \varphi }=\beta (1-p)\frac{\left(\delta -\gamma \right)}{\gamma \delta }<0, \end{aligned}$$

$$\begin{aligned} \frac{\partial {R}_{0}}{\partial \gamma } &=-\frac{\beta (1-p)\varphi }{{\gamma }^{2}}<0,\\ \frac{\partial {R}_{0}}{\partial \delta } &=-\frac{\beta \left[p{\eta}_{A}+\left(1-p\right)\left(1-\varphi \right)\right]}{{\delta }^{2}}<0,\\ \frac{\partial {R}_{0}}{\partial {\eta}_{A}} &=\frac{\beta p}{\delta }>0. \end{aligned}$$

These results suggest that ${R}_{0}$ is an increasing function of *β* and ${\eta}_{A}$, and a decreasing function of *φ*, *γ*, and *δ*. The effect of *p* on ${R}_{0}$ depends on the values of *φ*, *γ*, *δ*, and ${\eta}_{A}$. In other words, a higher transmission coefficient and a higher relative infectiousness of asymptomatic infections lead to a larger basic reproduction number, while a higher diagnosis proportion, a higher removal rate of diagnosed individuals, and a higher recovery rate of asymptomatic infected individuals and symptomatic but undiagnosed individuals reduce ${R}_{0}$. The corresponding sensitivity indices were calculated as follows:

$$\xi_\beta^{{\mathrm R}_0}=1,$$

$$\xi_{\mathrm P}^{{\mathrm R}_0}=\frac{\mathrm p\left(\frac{\eta_{\mathrm A}}\delta-\frac\varphi\gamma-\frac{1-\varphi}\delta\right)}{\frac{{\mathrm p\eta}_{\mathrm A}}\delta+\frac{\left(1-\mathrm p\right)\varphi}\gamma+\frac{\left(1-\mathrm p\right)\left(1-\varphi\right)}\delta},$$

$$\xi_\varphi^{{\mathrm R}_0}=\frac{\varphi\left(1-\mathrm p\right)\left(\frac1\gamma-\frac1\delta\right)}{\frac{{\mathrm p\eta}_{\mathrm A}}\delta+\frac{\left(1-\mathrm p\right)\varphi}\gamma+\frac{\left(1-\mathrm p\right)\left(1-\varphi\right)}\delta},$$

$$\xi_\gamma^{{\mathrm R}_0}=-\frac{\left(1-\mathrm p\right)\varphi/\gamma}{\frac{{\mathrm p\eta}_{\mathrm A}}\delta+\frac{\left(1-\mathrm p\right)\varphi}\gamma+\frac{\left(1-\mathrm p\right)\left(1-\varphi\right)}\delta},$$

$$\xi_\delta^{{\mathrm R}_0}=-\frac{\frac{{\mathrm{p}\upeta}_{\mathrm A}}\delta+\frac{\left(1-\mathrm p\right)\left(1-\varphi\right)}\delta}{\frac{{\mathrm p\eta}_{\mathrm A}}\delta+\frac{\left(1-\mathrm p\right)\varphi}\gamma+\frac{\left(1-\mathrm p\right)\left(1-\varphi\right)}\delta},$$

$$\xi_{\eta_{{}_{\mathrm A}}}^{{\mathrm R}_0}=-\frac{\frac{{\mathrm{p}\upeta}_{\mathrm A}}\delta}{\frac{{\mathrm p\eta}_{\mathrm A}}\delta+\frac{\left(1-\mathrm p\right)\varphi}\gamma+\frac{\left(1-\mathrm p\right)\left(1-\varphi\right)}\delta}.$$

Substituting the estimated parameter values into these expressions yielded the results summarized in Table 2.

**Table 2:** Sensitivity indices of the basic reproduction number

Parameter	Description	Sensitivity index
*β*	Transmission rate	1
*p*	Proportion of asymptomatic infections	−0.0493
*φ*	Proportion of diagnosed symptomatic cases	−0.2762
*γ*	Removal rate of diagnosed patients	−0.1381
*δ*	Recovery rate of undiagnosed symptomatic and asymptomatic infected individuals	−0.8619
${\eta}_{A}$	Relative infectiousness of asymptomatic infections	0.0924

## Transmission model with optimal control

Based on the factors influencing the transmission of mpox, and with reference to the *mpox Prevention and Control Plan* [14] and the *Core Information on Mpox Prevention and Control for Key Populations* [27], this study considers 4 control measures targeting the MSM population: (1) MSM community based prevention and control. (2) Early detection/Early treatment/Isolation. (3) Increasing medical consultation rate. (4) Vaccination. We extend Model (1) by introducing 4 variables to represent control measures and construct an mpox transmission dynamics model (3) with interventions to reduce infections and transmission. The 4 control variables are denoted by ${u}_{1}\mathrm{,}{u}_{2}{\text{, }u}_{3}{ \, and u}_{4}$. Each intervention contributes to mitigating mpox spread and plays an important role in controlling mpox incidence among the MSM population. The roles of these variables in mpox prevention and control are briefly summarized as follows:

1. ${u}_{1}$: MSM community based prevention and control, aiming to reduce high-risk behaviors and minimize the contact rate between susceptible individuals and infectious individuals, thereby lowering the overall transmission rate.
2. ${u}_{2}$: Early detection/Early treatment/Isolation, which shortens the time between symptom onset and isolation, thus limiting the number of secondary infections.
3. ${u}_{3}$: Increasing medical consultation rate, aiming to improve the rate at which symptomatic individuals seek medical care, thereby reducing undetected transmission in the MSM community.
4. ${u}_{4}$: Vaccination of susceptible individuals, which is assumed to confer complete protection during the control period, effectively removing vaccinated individuals from the susceptible population.

Additional assumptions and facts are incorporated in Model (3).

With MSM community based prevention and control in place, the transmission term is scaled by $(1-{u}_{1})$, so that susceptible individuals move into the exposed class at the reduced rate $(1-{u}_{1})\frac{\beta S({\eta}_{A}A+I+B)}{N}$ per unit time. Under Early detection/Early treatment/Isolation, the time to diagnosis decreases, and infectious individuals leave the infectious compartment at ${(1+u}_{2})\gamma I$ per unit time. For intervention increasing medical consultation rate, medical-seeking behavior among infected individuals improves, and the diagnosis rate is accordingly adjusted to ${(1+u}_{3})\varphi$*.* Vaccination acts on the susceptible population at rate ${\kappa u}_{4}$.*κ* is a scaling coefficient that converts ${u}_{4}$ into the effective transition rate. $\kappa {u}_{4}S$ vaccinated individuals transferring into the recovered compartment *R* per unit time, thereby reducing the number of susceptible individuals.

The system of ordinary differential equations for Model (3) is as follows:

3 $$\left\{\begin{array}{c}\frac{dS}{dt}=-(1-u_1)\frac{\beta S(\eta_AA+I+B)}N-\kappa u_4S\\\frac{dE}{dt}=(1-u_1)\frac{\beta S(\eta_AA+I+B)}N-p\omega E-(1-p){(1+u}_3)\varphi\omega E-\lbrack1-{(1+u}_3)\varphi\rbrack\left(1-p\right)\omega E\\\frac{dA}{dt}=p\omega E-\delta A\\\frac{dI}{dt}=(1-p){(1+u}_3)\varphi\omega E-{(1+u}_2)\gamma I\\\frac{dB}{dt}=\lbrack1-{(1+u}_3)\varphi\rbrack\left(1-p\right)\omega E-\delta B\\\frac{dR}{dt}={{\kappa u}_4S+(1+u}_2)\gamma I+\delta(A+B)\end{array}\right.$$

Based on the 4 control measures introduced in Model (3), various combinations of control measures were formulated to establish an integrated prevention and control framework. This framework was used to evaluate the effectiveness of mpox interventions and to identify the optimal control strategy and its implementation plan. To assess the impact of the 4 interventions and their combinations, four scenarios were designed according to the number of control measures implemented: single control measure, dual control measures, triple control measures, and quadruple control measures, denoted as Scenario A, Scenario B, Scenario C, and Scenario D, respectively. As shown in Table 3, each scenario consists of multiple schemes. For example, Scenario A (single control measure) includes 4 distinct schemes: MSM community based prevention and control only, Early detection/Early treatment/Isolation only, Increasing medical consultation rate only and Vaccination only, respectively. In total, 15 schemes were developed across all scenarios to assess the effectiveness of various interventions, with the aim of identifying the most efficient strategy to rapidly contain the spread of mpox.

**Table 3:** Control schemes

Scenario	Description	Implementation measure
Scenario A	Single control measure	${u}_{1}$≠0
		${u}_{2}$≠0
		${u}_{3}$≠0
		${u}_{4}$≠0
Scenario B	Dual control measures	${u}_{1}$,${u}_{2}$≠0
		${u}_{1}$,${u}_{3}$≠0
		${u}_{1}$,${u}_{4}$≠0
		${u}_{2}$,${u}_{3}$≠0
		${u}_{2}$,${u}_{4}$≠0
		${u}_{3}$,${u}_{4}$≠0
Scenario C	Triple control measures	${u}_{1}$,${u}_{2}$,${u}_{3}$≠0
		${u}_{1}$,${u}_{2}$,${u}_{4}$≠0
		${u}_{1}$,${u}_{3}$,${u}_{4}$≠0
		${u}_{2}$,${u}_{3}$,${u}_{4}$≠0
Scenario D	Quadruple control measures	${u}_{1}$,${u}_{2}$,${u}_{3}$,${u}_{4}$≠0

Based on the 15 mpox control schemes, this study conducts an optimal control analysis to evaluate interventions among the MSM. For each control scheme, the parameter values are expected to achieve the predefined control objectives while minimizing the associated costs. The ideal condition for optimal control in this study is to minimize the number of infected individuals, including those in the exposed (*E*), symptomatic and diagnosed (*I*), asymptomatic (*A*), and symptomatic but undiagnosed (*B*) classes, while reducing the cost of implementing the intervention measures.

Following the optimal control framework proposed by Pontryagin and the work of Fleming and Rishel [28], the control functions are assumed to be bounded and Lebesgue integrable. The admissible control set is defined as.

$$\begin{aligned} \mathrm{U} &=\{\left({u}_{1},{u}_{2},{u}_{3},{u}_{4}\right):0\le {u}_{i}\left(t\right)\le {u}_{i,\mathrm{m}\mathrm{a}\mathrm{x}},\\ & \quad t\in \left[0,{t}_{f}\right],i=\mathrm{1,2},\mathrm{3,4} \} \end{aligned}$$

The objective functional is given as:

4 $$\begin{aligned} J\left({u}_{1}{\mathrm{,}u}_{2}\mathrm{,}{u}_{3}{\mathrm{,}u}_{4}\right) = {\int}_{0}^{{t}_{f}} & \left(W\mathrm{+}\frac{{C}_{1}{u}_{1}^{2}}{2}+\frac{{C}_{2}{u}_{2}^{2}}{2}\right.\\ & \left.+\frac{{C}_{3}{u}_{3}^{2}}{2}+\frac{{C}_{4}{u}_{4}^{2}}{2}\right)dt \end{aligned}$$

where $$W={W}_{1}E\left(t\right)+{W}_{2}A\left(t\right)+{W}_{3}I\left(t\right)+{W}_{4}B(t)$$.

In the objective functional, $${W}_{i}, i=\mathrm{1,2},\mathrm{3,4}$$ are positive weighting constants associated with the state variables $$E\left(t\right), A\left(t\right), I\left(t\right)$$, and $$B\left(t\right)$$ respectively. The terms, $$\frac{{C}_{i}{u}_{i}^{2}}{2}, i=\mathrm{1,2},\mathrm{3,4}$$ represent the costs associated with implementing the control measures $${u}_{i}$$. The quadratic form reflects the increasing marginal costs of implementing the interventions and is strictly convex because $${C}_{i}$$>0. The coefficient 1/2 is included for algebraic convenience. The constants $${C}_{i}$$ denote the relative weight coefficients related to the cost of each control strategy. The objective is to determine the optimal control set ($${u}_{1}^{*},{u}_{2}^{*},{u}_{3}^{*},{u}_{4}^{*})$$ such that

$$J({u}_{1}^{*},{u}_{2}^{*},{u}_{3}^{*},{u}_{4}^{*})=\mathit{min}J({u}_{1}{,u}_{2},{u}_{3}{,u}_{4})$$

To obtain the optimal solution of the control model, the Lagrangian function is defined as follows:

$$\begin{aligned} & L\left({E, I, A, B, u}_{1}{,u}_{2},{u}_{3}{,u}_{4}\right)\\ &\quad ={W}_{1}E\left(t\right)+{W}_{2}A\left(t\right)+{W}_{3}I\left(t\right)+{W}_{4}B\left(t\right)\\ &\qquad+\frac{{C}_{1}{u}_{1}^{2}}{2}+\frac{{C}_{2}{u}_{2}^{2}}{2}+\frac{{C}_{3}{u}_{3}^{2}}{2}+\frac{{C}_{4}{u}_{4}^{2}}{2} \end{aligned}$$

According to Pontryagin’s Maximum Principle, the Hamiltonian is defined by adding the adjoint-weighted state equations to the running cost.

$$\begin{aligned} &H\left(S, E, A, I, B, R,{ u}_{i}{, \lambda }_{j}, t\right)\\ &\quad =L+{\lambda}_{1}\frac{dS}{dt}+{\lambda}_{2}\frac{dE}{dt}+{\lambda}_{3}\frac{dA}{dt}+{\lambda}_{4}\frac{dI}{dt}\\ & \qquad +{\lambda}_{5}\frac{dB}{dt}+{\lambda}_{6}\frac{dR}{dt} \end{aligned}$$

where ${\lambda}_{j}, j=1, 2, 3, 4, 5, 6$ are the adjoint variables corresponding to the state variables *S, E, A, I, B, R,* respectively.

The adjoint system and the optimality conditions are derived by taking the partial derivatives of the Hamiltonian with respect to state variables and control variables:

$$\begin{aligned} \frac{d{\lambda}_{1}}{dt} &=-\frac{\partial H}{\partial S},\frac{d{\lambda}_{2}}{dt}=-\frac{\partial H}{\partial E},\frac{d{\lambda}_{3}}{dt}=-\frac{\partial H}{\partial A},\\ \frac{d{\lambda}_{4}}{dt} &=-\frac{\partial H}{\partial I},\frac{d{\lambda}_{5}}{dt}=-\frac{\partial H}{\partial B},\frac{d{\lambda}_{6}}{dt}=-\frac{\partial H}{\partial R} \end{aligned}$$

Thus, the corresponding adjoint system is:

5 $$\begin{aligned} \frac{{d\lambda }_{1}}{dt} &\mathrm{=}\mathrm{(}{\lambda}_{1}-{\lambda}_{2}\mathrm{)}\mathrm{(}{1}-{u}_{1}\mathrm{)}\beta \frac{\mathrm{(}{\eta}_{\mathrm{A}}A\mathrm{+}I\mathrm{+}B\mathrm{)}}{N}\\ & \quad\mathrm{+}\mathrm{(}{\lambda}_{1}-{\lambda}_{6}\mathrm{)}\kappa {u}_{4}\\ \frac{{d\lambda }_{2}}{dt} &\mathrm{=}-{W}_{1}\mathrm{+}{\lambda}_{2}\omega -{\lambda}_{3}\omega p-\mathrm{(}{\lambda}_{4}-{\lambda}_{5}\mathrm{)}\mathrm{(}{1}-p\mathrm{)}\\ & \quad \mathrm{(}{1}\mathrm{+}{u}_{3}\mathrm{)}\varphi \omega -{\lambda}_{5}\mathrm{(}{1}-p\mathrm{)}\omega \\ \frac{{d\lambda }_{3}}{dt}&\mathrm{=}-{W}_{2}\mathrm{+}\mathrm{(}{\lambda}_{3}-{\lambda}_{6}\mathrm{)}\delta \mathrm{+}\mathrm{(}{\lambda}_{1}-{\lambda}_{2}\mathrm{)}\\ & \quad \mathrm{(}{1}-{u}_{1}\mathrm{)}\frac{\beta S{\eta}_{\mathrm{A}}}{N}\\ \frac{{d\lambda }_{4}}{dt}&\mathrm{=}-{W}_{3}\mathrm{+}\mathrm{(}{\lambda}_{4}-{\lambda}_{6}\mathrm{)}\mathrm{(}{1}\mathrm{+}{u}_{2}\mathrm{)}\gamma \mathrm{+}\mathrm{(}{\lambda}_{1}-{\lambda}_{2}\mathrm{)}\\ & \quad \mathrm{(}{1}-{u}_{1}\mathrm{)}\frac{\beta S}{N}\\ \frac{{d\lambda }_{5}}{dt}&\mathrm{=}-{W}_{4}\mathrm{+}\mathrm{(}{\lambda}_{5}-{\lambda}_{6}\mathrm{)}\delta \mathrm{+}\mathrm{(}{\lambda}_{1}-{\lambda}_{2}\mathrm{)}\\ & \quad \mathrm{(}{1}-{u}_{1}\mathrm{)}\frac{\beta S}{N}\\ \frac{{d\lambda }_{6}}{dt}&\mathrm{=}{0}\end{aligned}$$

with the transversality conditions

$${\lambda}_{j}\left({t}_{f}\right)=0, j=\mathrm{1,2},\mathrm{3,4},\mathrm{5,6}.$$

Since $\frac{\partial H}{\partial {u}_{i}}=0, i=\mathrm{1,2},\mathrm{3,4}$, we have.

$$\begin{aligned} &{u}_{1}^{*}\mathrm{=}\frac{\mathrm{(}{\lambda}_{2}-{\lambda}_{1}\mathrm{)}\beta S\mathrm{(}{\eta}_{A}A\mathrm{+}I\mathrm{+}B\mathrm{)}}{{C}_{1}N}\mathrm{,}{ \, u}_{2}^{*}\mathrm{=}\frac{\mathrm{(}{\lambda}_{4}-{\lambda}_{6}\mathrm{)}\gamma I}{{C}_{2}}\mathrm{, }\\ &{u}_{3}^{*}\mathrm{=}\frac{\mathrm{(}{\lambda}_{4}-{\lambda}_{5}\mathrm{)(}p\mathrm{-1)}\varphi \omega E}{{C}_{3}}\mathrm{,}{ \, u}_{4}^{*}\mathrm{=}\frac{\kappa \mathrm{(}{\lambda}_{1}-{\lambda}_{6}\mathrm{)}S}{{C}_{4}}. \end{aligned}$$

Thus

$${u}_{1}^{*}(t)=\mathit{min}\left({u}_{1,max},\mathit{max}\left(0,\frac{\left({\lambda}_{2}-{\lambda}_{1}\right)\beta S\left({\eta}_{A}A+I+B\right)}{{C}_{1}N}\right)\right),$$

$${u}_{2}^{*}(t)=\mathit{min}\left({u}_{2,max},\mathit{max}\left(0,\frac{\left({\lambda}_{4}-{\lambda}_{6}\right)\gamma I}{{C}_{2}}\right)\right),$$

$${u}_{3}^{*}(t)=\mathit{min}\left({u}_{3,max},\mathit{max}\left(0,\frac{\left({\lambda}_{5}-{\lambda}_{4}\right)\left(1-p\right)\varphi \omega E}{{C}_{3}}\right)\right),$$

$${u}_{4}^{*}(t)\mathrm{=}{\mathrm{min}}\left({u}_{\mathrm{4,}max}\mathrm{,}{\mathrm{max}}\left(\mathrm{0,}\frac{\kappa \left({\lambda}_{1}-{\lambda}_{6}\right)S}{{C}_{4}}\right)\right),$$

To evaluate the impact of various optimal control strategies, we simulate four scenarios (including 15 schemes) to assess the effectiveness of mpox containment among the MSM population in the central Chinese city. Optimal control is initiated 20 days after the onset of mpox transmission, coinciding with heightened societal and medical attention. In the optimal control simulations, the upper bounds of the four control variables were set as *u*_1,max_ = *u*_2,max_ = *u*_3,max_ = *u*_4,max_ = 0.8. The variable *u*_4_ represents the vaccination control intensity. The effective vaccination transition rate is *κu*_4_, where *κ* = 0.02; therefore, the maximum effective daily vaccination transition rate is 0.016. (see Table 4). The positive weight constants in the objective function are assumed equal ${W}_{i}\text{=1, }i\text{=1, 2, 3, 4}$ [29–32]. Simultaneously, weight constants associated with the overall implementation costs are assumed unequal, reflecting the unequal costs of the 4 control measures: MSM community based prevention and control, Early detection/Early treatment/Isolation, Increasing medical consultation rate and Vaccination, denoted as ${C}_{1}=40,{ C}_{2}=50, {C}_{3}=20,{ C}_{4}=100$ [29–32]. The simulation duration is 100 days; accordingly, ${t}_{f}$=100. Numerical simulations are carried out using Python, demonstrating the temporal dynamics of mpox transmission under varying control conditions and quantifying population compartment changes.

**Table 4:** Bounds for control measures representing available effort

Control	Definition	Bounds
${u}_{1}$	MSM community based prevention and control	[0,0.8]
${u}_{2}$	Early detection/Early treatment/Isolation	[0,0.8]
${u}_{3}$	Increasing medical consultation rate	[0,0.8]
${u}_{4}$	Vaccination	[0,0.8]

## Transmission model with constant control

We further perform simulations under constant quadruple control measures to compare with the optimal control outcomes under Scenario D. The constant control settings are constructed such that, within each group, only one control variable is varied while the remaining controls are fixed at a moderate level, defined as 62.5% of their upper bounds used in the optimal control ranges. For instance, in Group I, ${u}_{1}$ is set to 0.2, 0.4, 0.6 and 0.8, whereas ${u}_{2}$=0.5 ${u}_{3}$=0.5 ${u}_{4}$=0.5 are fixed. As summarized in Table 5, four groups denoted as Groups I–IV are simulated to examine how different constant levels of each control measure affect mpox transmission under quadruple constant control.

**Table 5:** Simulation settings under constant quadruple control measures

Group	Varied control variable with levels	Fixed values of the other controls
Group I	${u}_{1}$=0.2, 0.4, 0.6, 0.8	${u}_{2}$=0.5 ${u}_{3}$=0.5 ${u}_{4}$=0.5
Group Ⅱ	${u}_{2}$=0.2, 0.4, 0.6, 0.8	${u}_{1}$=0.5 ${u}_{3}$=0.5 ${u}_{4}$=0.5
Group Ⅲ	${u}_{3}$=0.2, 0.4, 0.6, 0.8	${u}_{1}$=0.5 ${u}_{2}$=0.5 ${u}_{4}$=0.5
Group Ⅳ	${u}_{4}$=0.2, 0.4, 0.6, 0.8	${{u}_{1}=0.5 u}_{2}$=0.5 ${u}_{3}$=0.5

## Parameter values

In this study, the size of the MSM population in a central city was estimated using a proportional estimation method. According to the literature, the proportion of MSM individuals among sexually active males in China ranges from 2 to 4% [33]. Based on previous studies conducted in the same city and in other cities with comparable population sizes, the proportion of MSM individuals among males aged 15–59 years was estimated to be approximately 2%–2.5% [34–36]. According to the Seventh National Census, the total number of males aged 15–59 years in the city was 3,448,714 [37]. Consequently, the estimated size of the MSM population in the city ranges from approximately 69,000 to 86,000 individuals. Accordingly, the MSM population size (*N*) was set to 81,224 in this study, corresponding to an estimated proportion of 2.35% among males aged 15–59 years.

Drawing upon this population estimate, the natural history of mpox, and the local epidemiological context, a compartmental transmission model for mpox was constructed. The model assumes that mpox infections in this city are confined to the MSM population. All reported cases were detected through voluntary medical consultation, with mild clinical presentations predominantly occurring among young and middle-aged adult males, all of whom reported recent MSM activity prior to symptom onset. The latent period for mpox typically ranges from 5 to 21 days, with a common duration of 6 to 13 days [38]. Local epidemiological data suggest a latent period ranging from 4.25 to 7 days, with a median of 5 days [39]. Accordingly, the model adopts a latent period of 5 days, corresponding to a daily latent rate of 1/5 = 0.2.

Given that mpox is primarily transmitted through sexual contact, and that MSM individuals often refrain from seeking medical care due to perceiving their symptoms as non-severe or unnecessary to report [40], the medical consultation rate was set at 35%. The proportion of asymptomatic mpox cases is estimated to slightly exceed 13%, which is comparable to SARS [41]; therefore, the initial proportion of asymptomatic cases was set to 13.5%. The interval from symptom onset to medical consultation ranges from 4 to 9.5 days, with a median of 6.88 days [39]; thus, the daily removal rate from the diagnosed symptomatic compartment was set as 1/6.88≈0.15. For symptomatic individuals who do not seek care, as well as asymptomatic individuals, the duration of illness ranges from 2 to 4 weeks [42]. Assuming an average duration of 3 weeks, the natural recovery rate was set to 1/21≈0.05. Direct empirical estimates of the relative infectiousness of asymptomatic and diagnosed symptomatic mpox infections remain limited. Therefore, symptomatic but undiagnosed individuals were used as the reference group, while asymptomatic infected individuals were assumed to have lower infectiousness due to the absence of typical lesions or symptoms, and *η*_*A*_ was set to 0.5 [43].

Previous studies suggest that the basic reproduction number (*R*_*0*_) of mpox among MSM populations ranges from 1.5 to 2.3 [44–47]. In this study, we assume an *R*_*0*_ of 1.8 for the MSM population in the city. The transmission rate (*β*) was computed using the next-generation matrix method based on the assumed *R*_*0*_. The parameter values adopted in the model are summarized in Table 1.

## Results

### Simulation of Mpox transmission without control measures

**Fig. 2:**
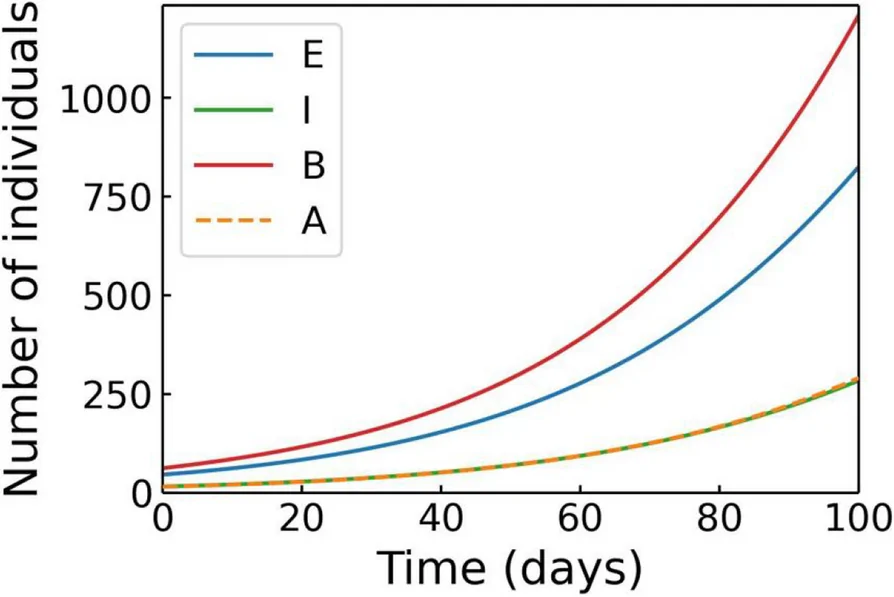
Mpox transmission simulation without control measures

In the absence of any control measures, the population sizes in each compartment of the mpox dynamics undergo prolonged temporal changes. Figure 2 presents the time evolution over 100 days of the exposed (*E*), asymptomatic (*A*), symptomatic and diagnosed (*I*) and symptomatic but undiagnosed (*B*) under setting without control. Without control measures, the simulation generated a total of 6331 infections within 100 days.

## Simulation of Mpox transmission with control measures

### Optimal control strategies for Mpox

In this section, we compared mpox transmission over a 100-day period under the setting without control and 15 optimal control schemes. Without control, the cumulative number of infections reached 6331 by day 100, whereas the implementation of any control scheme was associated with a marked reduction in both the symptomatic and diagnosed *I*(*t*) and the total number of infections.

**Fig. 3:**
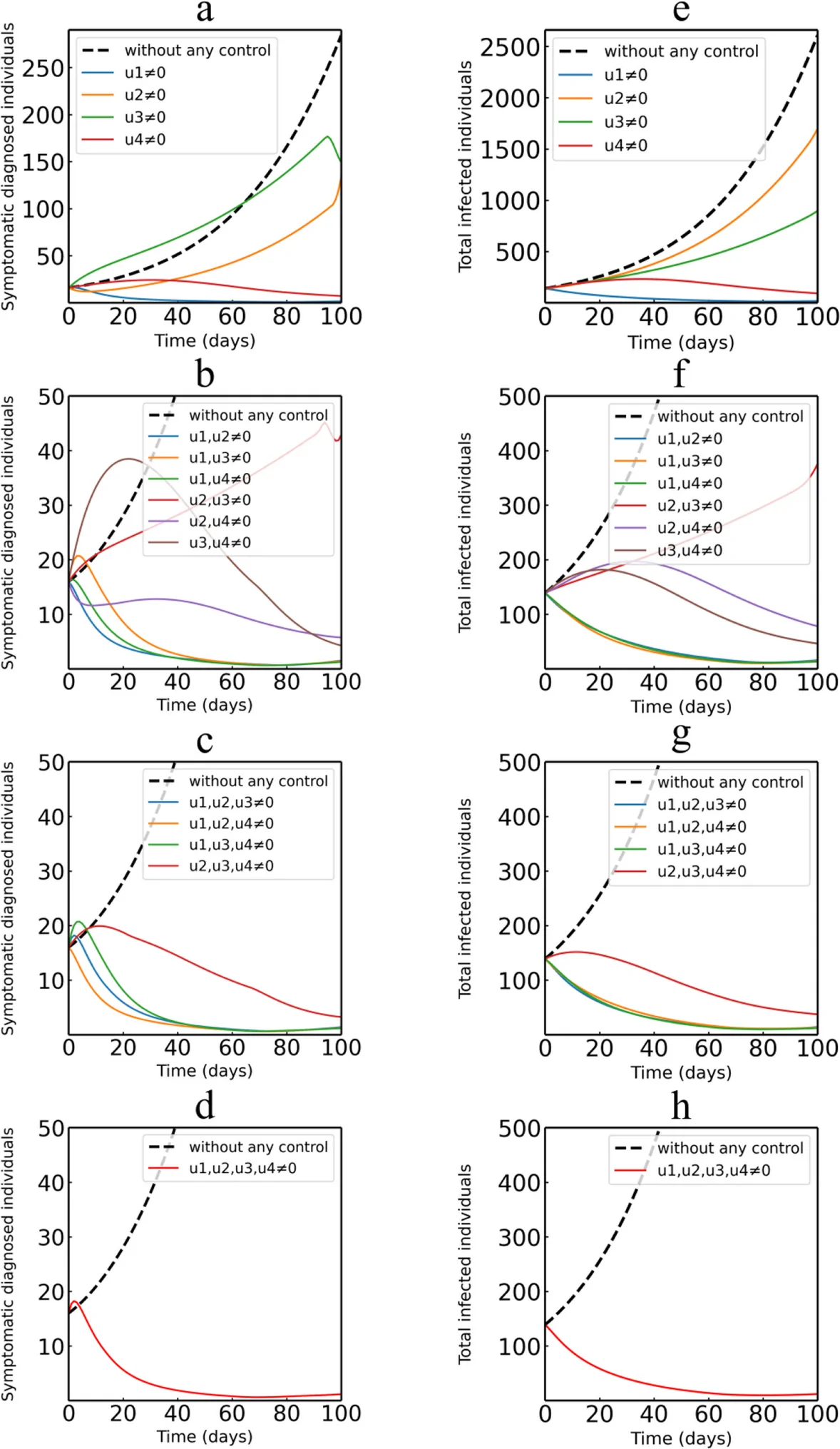
The effect of implementing control measures of four scenarios. Panels **a**–**d** show the temporal variation in the number of symptomatic and diagnosed individuals *I(t)*; panels e–h show the temporal variation in the total number of infected individuals (*E(t)* + *A(t)* + *I(t)* + *B(t)*)

- Scenario A (single control measure) shows distinct time-course patterns of symptomatic diagnosed infections *I*(*t*) and total infections across the 4 single interventions (Fig. 3a and e). Under *u*_1_ or *u*_4_ alone, *I*(*t*) remains at low levels during the mid-to-late period, and the accumulation of total infections increases slowly over time. Under *u*_2_ alone, *I(t)* continues to increase during the middle and later phases, while total infections keep accumulating throughout the period. Under *u*_3_ alone, *I(t)* is higher than without control during the early period and subsequently declines, whereas total infections continue to increase over time (Fig. 3a and e). Overall, *u*_1_ achieves the best performance among single control measures, averting 6238 infections.
- Scenario B (dual control measures) presents further separation in trajectories of *I*(*t*) and total infections across different pairs (Fig. 3b and f). Combinations including *u*_1_ (*u*_1_ + *u*_2_, *u*_1_ + *u*_3_, *u*_1_ + *u*_4_) display *I*(*t*) maintained near low levels for most of the period. Combinations including *u*_4_ (*u*_2_ + *u*_4_, *u*_3_ + *u*_4_) similarly show low *I*(*t*) and slower accumulation of total infections. In contrast, the *u*_2_ + *u*_3_ pairing exhibits non-zero *I*(*t*) in the mid-to-late phase, accompanied by continued increases in total infections over time (Fig. 3b and f). Under scenario B, *u*_1_ + *u*_4_ achieves the strongest control effect, averting 6250 infections (Fig. 3f).
- Scenario C (triple control measures) indicates that, across all three-way combinations, both *I*(*t*) and total infections decline rapidly early and remain near zero thereafter, with only small differences among the alternative triple-control sets (Fig. 3c and g). The most effective combination in scenario C is *u*_1+_*u*_*3*+_*u*_*4*_, which averts 6256 infections (Fig. 3g).
- Scenario D (quadruple control measures) shows that *I*(*t*) drops to near zero early and stays at very low level across the entire simulation window, while total infections are also maintained at very low levels with trajectories close to the baseline axis (Fig. 3d and h). Under Scenario D, a total of 6258 infections are averted (Fig. 3h). Results for all 15 optimal control schemes are provided in Additional file 1: Table S1.

**Fig. 4:**
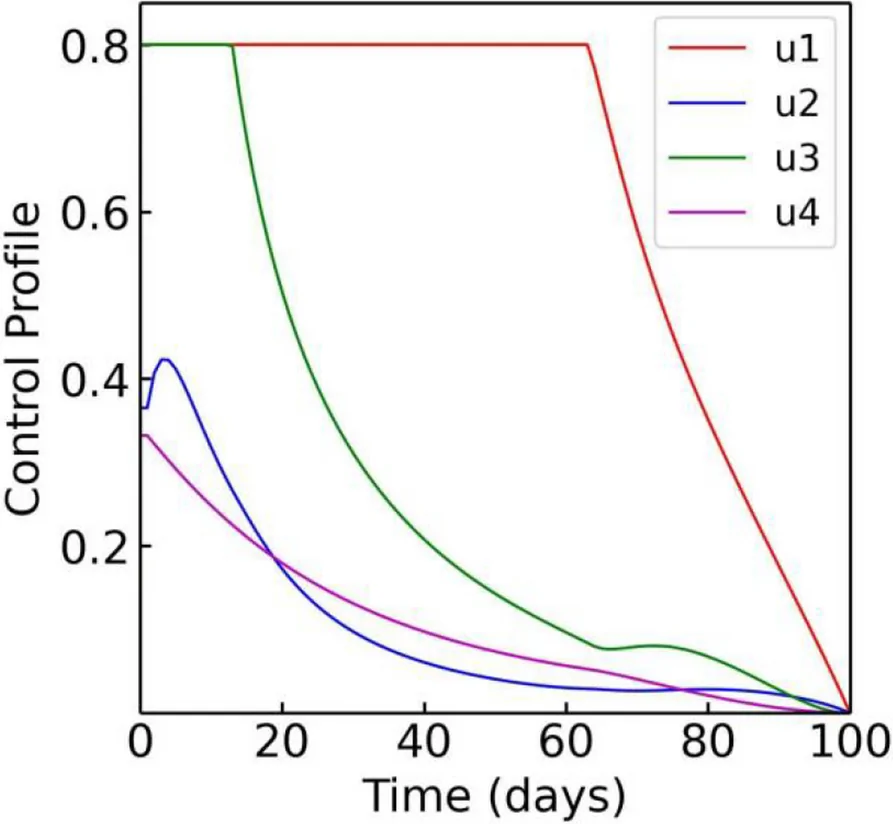
Optimal control for the mpox of scenario D

The corresponding optimal control profiles (*u*_*1*_,*u*_*2*_,*u*_*3*_,*u*_*4*_) of Scenario D are shown in Fig. 4 (See Additional file 1:Fig. S1−3 for control profiles of Scenario A, B, C). The control *u*_*1*_ is maintained near its upper bound for an extended initial interval and then tapers down toward zero approaching day 100, while *u*_*3*_ starts at a high level and decreases rapidly during the early-to-mid period. The control *u*_*2*_ displays an early peak followed by a monotone decline, and *u*_*4*_ remains at its upper bound during the early control period, promoting the transfer of susceptible individuals to the recovered compartment through vaccination, and then gradually declines to near-zero levels in the later period.

### Constant control for Mpox

**Fig. 5:**
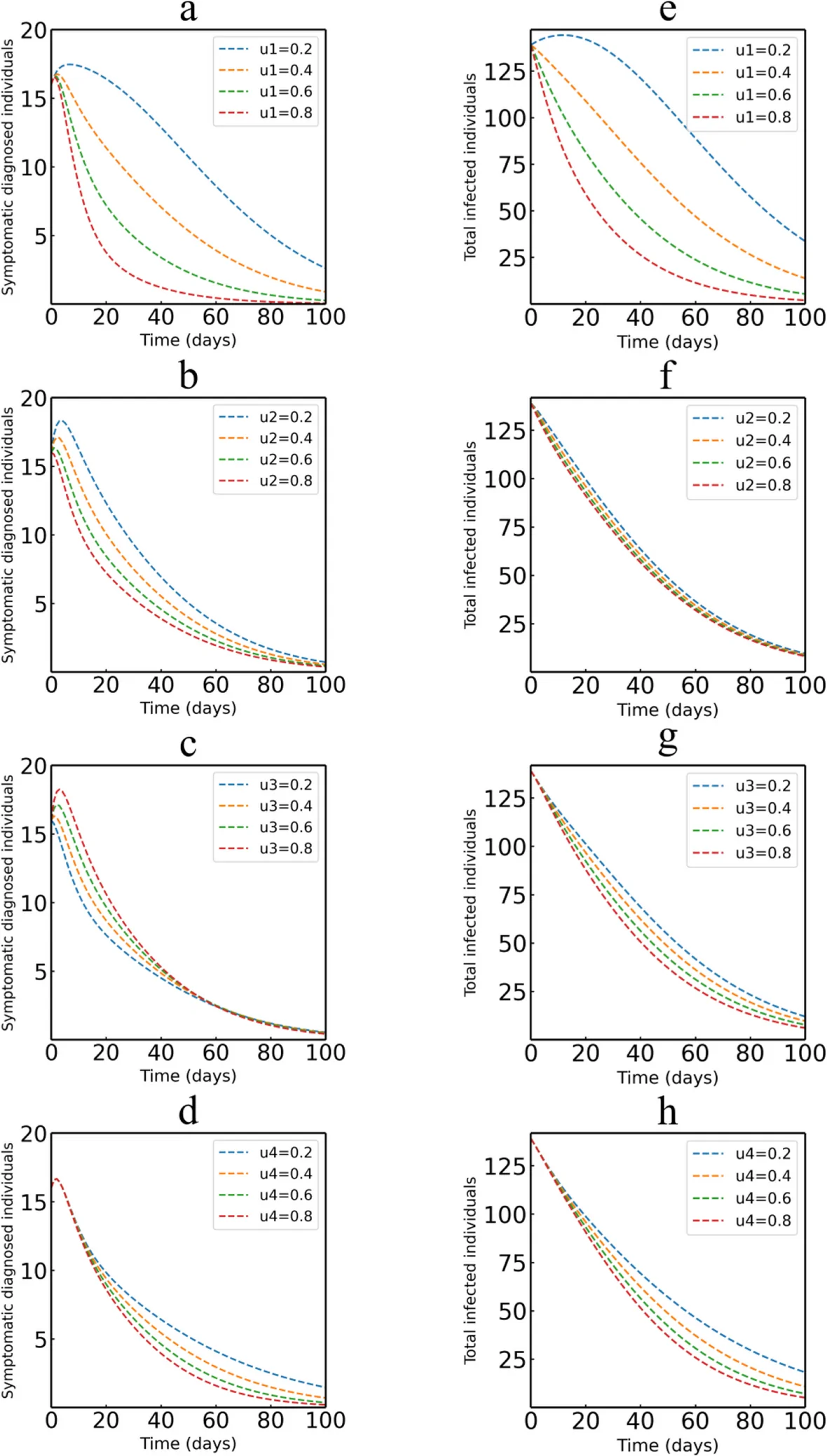
The effect of implementing constant quadruple control measures. Panels **a**–**d** show the temporal variation in the number of symptomatic and diagnosed individuals *I(t)*; panels **e**–**h** show the temporal variation in the total number of infected individuals (*E(t)* + *A(t)* + *I(t)* + *B(t)*)

Figure 5 illustrates the simulated transmission outcomes of mpox over 100 days under four groups of constant control settings. In Group I, *u*_*1*_ varies while *u*_*2*_, *u*_*3*_, and *u*_*4*_ are held constant. Within this group, changes in *u*_*1*_ exert a pronounced influence on symptomatic diagnosed infections and total infections (Fig. 5a and e). When *u*_*1*_ = 0.8, this constant-control setting averts 6280 infections.

In Group II, variation in *u*_*2*_ substantially affects symptomatic diagnosed infections (Fig. 5b), whereas its impact on total infections is relatively modest (Fig. 5f). When *u*_*2*_ = 0.8, this constant control setting averts 6154 infections.

In Group III, changes in *u*_*3*_ have a stronger effect on symptomatic diagnosed infections during the early stage of control. Notably, larger values of *u*_*3*_ are associated with more symptomatic diagnosed infections in the early phase (Fig. 5c). For total infections, increasing *u*_*3*_ leads to a more noticeable reduction in the later period, although the overall effect remains comparatively mild (Fig. 5g). When *u*_*3*_ = 0.8, this constant-control setting averts 6165 infections.

In Group IV, the effect of varying *u*_*4*_ on both symptomatic diagnosed infections and total infections is relatively small in the early period, but gradually becomes more evident in the later period. When *u*_*4*_ = 0.8, this constant control setting averts 6181 infections (Fig. 5d and h). Results under all constant settings in Groups I–IV are summarized in Additional file 1: Table S2.

### Advantage of optimal control

**Fig. 6:**
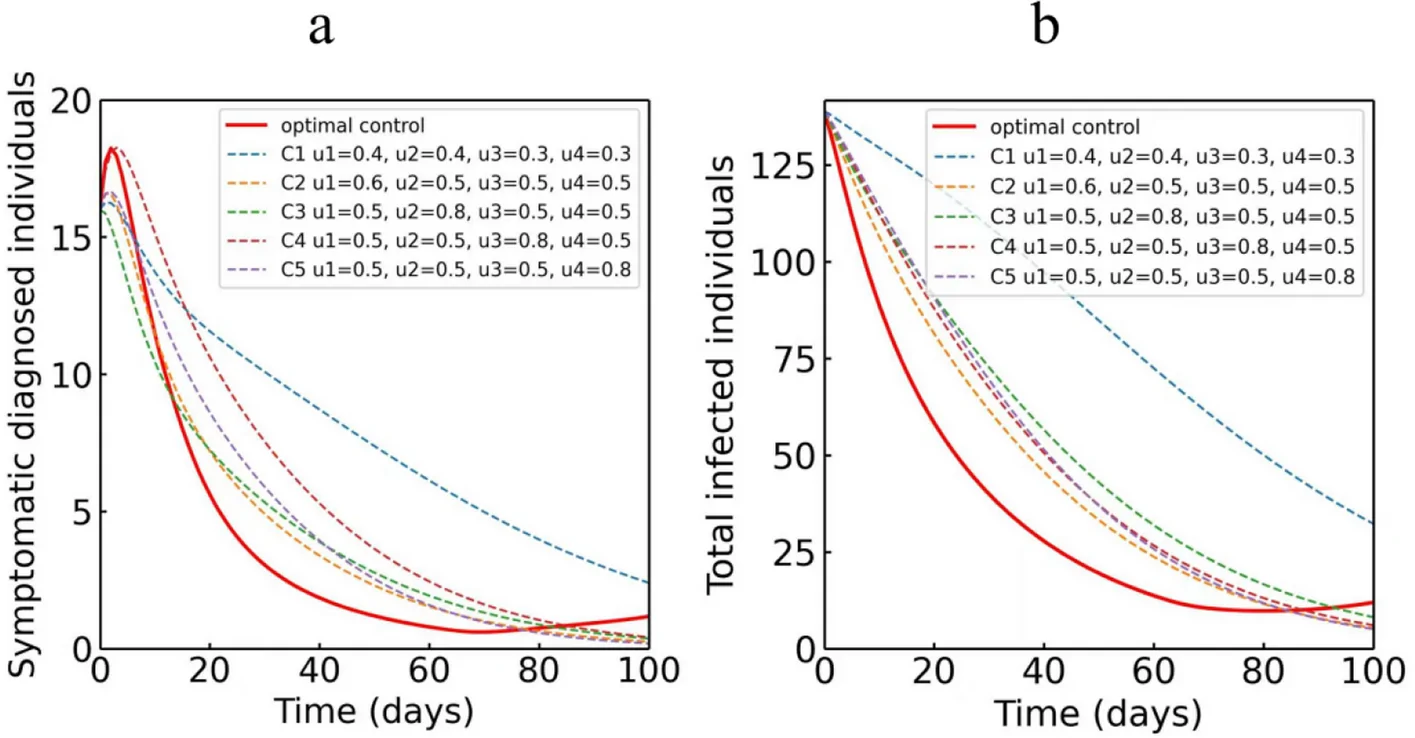
Comparison of optimal control and constant control strategies. Panel **a** shows the temporal variation in the number of symptomatic and diagnosed individuals *I(t)*; panel **b** shows the temporal variation in the total number of infected individuals (*E(t)* + *A(t)* + *I(t)* + *B(t)*)

Figure 6 Comparison of optimal control and constant control strategies presents the comparison between the optimal control and five constant control settings (C1–C5). Specifically, C1 is a relatively balanced, low-intensity combination of the 4 constant control measures, whereas C2–C5 are four constant control combinations selected from Fig. 5 that are close to the optimal control performance under Scenario D

Across both outcome measures, symptomatic diagnosed individuals and total infections, all controlled trajectories decline over time relative to their initial levels, with distinct rates of decrease under different strategies (Fig. 6a–b). Under the optimal control, the decline is steep during the early phase and the curves approach low levels by the late phase of the 100-day period. By contrast, constant controls show heterogeneous patterns that depend on the fixed intensities of (*u*_*1*_,*u*_*2*_,*u*_*3*_,*u*_*4*_). In particular, the setting with lower fixed intensities (C1: *u*_*1*_ = 0.4, *u*_*2*_ = 0.4, *u*_*3*_ = 0.3, *u*_*4*_ = 0.3) exhibits a slower decrease and a higher residual level at day 100, whereas higher-intensity settings (C2–C5) yield trajectories closer to those of the optimal control, with earlier reductions and lower terminal levels. The constant control settings C1–C5 avert 5980, 6202, 6154, 6165, and 6181 infections, respectively.

Control expenditures are summarized using the quadratic “control-only” cost term, *C*_*1*_*u*_*1*_^*2*^/2 + *C*_*2*_*u*_*2*_^*2*^/2 + *C*_*3*_*u*_*3*_^*2*^/2 + *C*_*4*_*u*_*4*_^*2*^/2 accumulated over the 100-day period. The optimal control yields Control-only cost = 1209.57 (dimensionless). For constant controls, the corresponding total control costs over 100 days are C1: 1260.00, C2: 2845.00, C3: 3600.00, C4: 3015.00, and C5: 4575.00, providing the cost range associated with time-independent implementations. Results under C1–C5 and quadruple optimal control are summarized in Additional file 1: Table S3. Results of sensitivity analyses assessing the robustness of the optimal control conclusions are provided in Additional file 2.

## Discussion

This study developed a compartmental transmission dynamics model for mpox in the MSM population of a central Chinese city and incorporated optimal control to evaluate coordinated intervention strategies. By explicitly representing asymptomatic infections (*A*), with relative infectiousness described by *η*_*A*_, and symptomatic but undiagnosed infections (*B*), the framework captures transmission that may occur outside routine case detection, while symptomatic and diagnosed infections (*I*) describe the clinically identified burden. In the numerical simulations without controls, infections increased over time, indicating the potential for sustained transmission in the MSM population and supporting the need for timely interventions.

The scenario based optimal control analysis considered 4 measures consistent with routine prevention and control practice: MSM community based prevention and control (*u*_*1*_), Early detection/Early treatment/Isolation (*u*_*2*_), increasing medical consultation rate (*u*_*3*_), and vaccination (*u*_*4*_). The simulations show that different intervention sets generate clearly different trajectories for symptomatic diagnosed infections and total infections. The observation is consistent with practical experience that implementing a single control measure is usually insufficient for outbreak control in MSM communities, and it is also consistent with the fact that a single optimal control profile may be difficult to achieve in routine work. For example, when implemented alone, increasing medical consultation rate (*u*_*3*_) may increase symptomatic diagnosed infections in the early period, which should be interpreted as more symptomatic individuals moving from *B(t)* to *I(t)* rather than as higher transmission. In fact, *u*_3_ reduces total infections continuously. However, the temporary increase in symptomatic diagnosed infections may impose additional pressure on healthcare services, whereas the increase can be attenuated when *u*_*3*_ is combined with other measures. Therefore, mpox control typically requires combining multiple measures, and the quadruple control measure is used as a practical representation of an operational best scheme.

Scenario D (quadruple control measures) represents the most comprehensive intervention set among the scenarios. Under Scenario D, symptomatic diagnosed infections *I(t)* decline rapidly in the early stage and remain at very low levels over the control period, and total infections are maintained at a low level as well. The model structure provides a mechanistic interpretation for this pattern. Community based prevention and control (*u*_*1*_) reduces effective contacts within MSM networks and lowers the force of infection; Early detection/Early treatment/Isolation (*u*_*2*_) accelerates movement out of infectious states by shortening the time from symptom onset to isolation; increasing medical consultation rate (*u*_*3*_) reduces the size and duration of the symptomatic but undiagnosed compartment (*B*) that can drive hidden transmission; vaccination (*u*_*4*_), through the effective transition rate *κu*_*4*_, transfers susceptible individuals to the recovered compartment and limits onward spread. Because these controls act on different points of the transmission process, their simultaneous implementation can suppress both observed cases and the unobserved infectious pool.

This study further compared time-dependent optimal control with time-independent constant control under the quadruple control setting. It is observed that constant controls lead to different decline patterns depending on fixed intensity levels, whereas the optimal strategy typically allocates stronger effort earlier and then gradually reduces intensity as infections decline. In operational terms, this corresponds to concentrating resources at the start of response, including intensified community engagement and risk communication, expanded testing and rapid isolation, and targeted vaccination, followed by adjustment of intensities as the epidemic burden decreases. The model formulation also clarifies the role of *u*_*3*_ in shaping reported case curves. By increasing medical consultation, *u*_*3*_ shifts symptomatic infections from the undiagnosed compartment into the diagnosed compartment, which can change the temporal pattern of *I(t)* even when total infections decline, and this is relevant for interpreting surveillance data during response. Moreover, the constant control experiments provide a simple proxy for fixed program intensity, such as maintaining a stable level of community intervention or vaccination. The comparison suggests that selecting constant levels that are too low may leave a prolonged tail of infections, while selecting levels that are too high may increase accumulated cost without proportional benefit. Therefore, the optimal control profiles can be used to propose time schedules for implementing *u*_*1*_ to *u*_*4*_, including an early period of higher intensity followed by gradual relaxation, which is consistent with adaptive public health management.

Several limitations should be acknowledged. The model neglects demographic processes and disease induced mortality, which may influence projections on longer periods beyond the control window. The MSM population is heterogeneous in contact behavior, and homogeneous mixing may underestimate the contribution of high activity subgroups; further work could stratify by activity level or incorporate contact structure. Parameter values are partly assumed and partly drawn from available information; future studies should calibrate to local surveillance and behavioral data and quantify uncertainty. The relative infectiousness parameter *η*_*A*_ was specified using common model assumptions because direct empirical estimates remain limited. In addition, vaccine protection, supply constraints, and delayed onset of immunity were simplified, and incorporating these elements would improve realism. Despite these limitations, the model provides a coherent framework for examining integrated control strategies and supports the use of Scenario D and time-dependent optimal control as informative references for mpox prevention and control in MSM communities.

## Conclusions

To characterize the spread of mpox in a city, we developed an mpox transmission dynamics model. In addition, we considered 4 control measures and applied optimal control theory to identify the best control strategy. Our study finds that, in the absence of control measures, mpox can result in a substantial cumulative infections among MSM in a city. Implementing any control measures reduces the total infections. Symptomatic diagnosed infections generally decrease under most control measures, they also reflect more symptomatic individuals being diagnosed when the medical consultation rate is increased. MSM community based prevention and control (*u*_1_) is the best single control measure. Quadruple control measures which include MSM community based prevention and control, Early detection/Early treatment/Isolation, Increasing medical consultation rate and Vaccination, averted the largest number of infections (6258, 98.8%). The optimal control profiles allocate stronger efforts in the initial stage and then reduce the intensity gradually over time. Compared with constant control measures, optimal control achieves higher effectiveness at comparable cost.

## Supplementary Information

Additional file 1: Summary tables of control simulation results and optimal control profiles. 

Additional file 2: Sensitivity analyses of transmission dynamics model parameters and control measures.

## Data Availability

The data that support the findings of this study are available in this article, and the code of the study can be accessed by contacting the corresponding author.

## Abbreviations

MPXV: Monkeypox virus
WHO: World Health Organization
PHEIC: Public Health Emergency of International Concern
COVID-19: Coronavirus disease 2019
MSM: Men who have sex with men
${R}_{0}$: Basic reproduction number
