# Supplementary material for: Dynamic modeling and optimal control of Mpox among MSM in a central Chinese city
Source: BMC Public Health. 2026 Aug 15;26:3351. doi: 10.1186/s12889-026-28925-6 (PMC13753281; doi:10.1186/s12889-026-28925-6)
Supplement: Supplementary file 1 [file 12889_2026_28925_MOESM1_ESM.docx]

**Additional file 1**

**Dynamic modeling and optimal control of Mpox among MSM in a central Chinese city**

Dan Wang, Si Wu, Siyu Zhang, Han Ni, Yuxi He, Liuyi Fu, Jin Zhao, Yuan Lv, Wenting Zha

**Contents**

[Summary tables for control simulation results...........................................1](#_Toc928)

[Control profiles for Scenario A-D (single, dual, triple, and quadruple control measures)........................................................................................4](#_Toc21998)

**Summary tables for control simulation results**

| Scenario | Description | Implementation measure | Total infections | Infections averted | Control-only cost | Infections averted rank |
| --- | --- | --- | --- | --- | --- | --- |
| Scenario A | Single control measure | *u*_1_≠0 | 93 | 6238 | 1041.02 | 8 |
|  |  | *u*_2_≠0 | 4535 | 1796 | 1563.71 | 15 |
|  |  | *u*_3_≠0 | 3061 | 3270 | 609.99 | 14 |
|  |  | *u*_4_≠0 | 804 | 5527 | 2002.69 | 12 |
| Scenario B | Dual control measures | *u*_1_,*u*_2_≠0 | 91 | 6240 | 1081.29 | 7 |
|  |  | *u*_1_,*u*_3_≠0 | 83 | 6248 | 1152.86 | 6 |
|  |  | *u*_1_,*u*_4_≠0 | 81 | 6250 | 1088.27 | 5 |
|  |  | *u*_2_,*u*_3_≠0 | 1675 | 4656 | 2116.56 | 13 |
|  |  | *u*_2_,*u*_4_≠0 | 701 | 5630 | 2573.26 | 11 |
|  |  | *u*_3_,*u*_4_≠0 | 593 | 5738 | 2144.46 | 10 |
| Scenario C | Triple control measures | *u*_1_,*u*_2_,*u*_3_≠0 | 80 | 6251 | 1209.70 | 4 |
|  |  | *u*_1_,*u*_2_,*u*_4_≠0 | 79 | 6252 | 1120.11 | 3 |
|  |  | *u*_1_,*u*_3_,*u*_4_≠0 | 75 | 6256 | 1161.51 | 2 |
|  |  | *u*_2_,*u*_3_,*u*_4_≠0 | 475 | 5856 | 2630.41 | 9 |
| Scenario D | Quadruple control measures | *u*_1_,*u*_2_,*u*_3_,*u*_4_≠0 | 73 | 6258 | 1209.57 | 1 |

Table S1 Summary of results under the 15 optimal control schemes

Total infections indicate the cumulative number of infections. Infections averted are calculated relative to the scenario without control, in which cumulative infections reach 6331. Control-only cost represents the accumulated cost of implementing each control scheme. Infections averted rank is determined according to the number of infections averted.

| Group | Fixed controls | Varied control variable | Control level | Total infections | Infections averted | Control-only cost |
| --- | --- | --- | --- | --- | --- | --- |
| Group I | *u*_2_=*u*_3_=*u*_4_=0.5 | *u*_1_ | 0.2 | 458 | 5873 | 2205.00 |
|  |  |  | 0.4 | 254 | 6077 | 2445.00 |
|  |  |  | 0.6 | 129 | 6202 | 2845.00 |
|  |  |  | 0.8 | 51 | 6280 | 3405.00 |
| Group II | *u*_1_=*u*_3_=*u*_4_=0.5 | *u*_2_ | 0.2 | 194 | 6137 | 2100.00 |
|  |  |  | 0.4 | 187 | 6144 | 2400.00 |
|  |  |  | 0.6 | 181 | 6150 | 2900.00 |
|  |  |  | 0.8 | 177 | 6154 | 3600.00 |
| Group III | *u*_1_=*u*_2_=*u*_4_=0.5 | *u*_3_ | 0.2 | 203 | 6128 | 2415.00 |
|  |  |  | 0.4 | 190 | 6141 | 2535.00 |
|  |  |  | 0.6 | 178 | 6153 | 2735.00 |
|  |  |  | 0.8 | 166 | 6165 | 3015.00 |
| Group IV | *u*_1_=*u*_2_=*u*_3_=0.5 | *u*_4_ | 0.2 | 242 | 6089 | 1575.00 |
|  |  |  | 0.4 | 199 | 6132 | 2175.00 |
|  |  |  | 0.6 | 171 | 6160 | 3175.00 |
|  |  |  | 0.8 | 150 | 6181 | 4575.00 |

Table S2 Summary of results under constant quadruple control groups

In each group, one control variable varies and the other three are fixed at 0.5. Total infections indicate the cumulative number of infections. Infections averted are calculated relative to the scenario without control, in which cumulative infections reach 6331. Control-only cost represents the accumulated cost of each setting.

| Setting | *u*_1_ | *u*_2_ | *u*_3_ | *u*_4_ | Total infections | Infections averted | Control-only cost |
| --- | --- | --- | --- | --- | --- | --- | --- |
| C1 | 0.4 | 0.4 | 0.3 | 0.3 | 351 | 5980 | 1260.00 |
| C2 | 0.6 | 0.5 | 0.5 | 0.5 | 129 | 6202 | 2845.00 |
| C3 | 0.5 | 0.8 | 0.5 | 0.5 | 177 | 6154 | 3600.00 |
| C4 | 0.5 | 0.5 | 0.8 | 0.5 | 166 | 6165 | 3015.00 |
| C5 | 0.5 | 0.5 | 0.5 | 0.8 | 150 | 6181 | 4575.00 |
| Quadruple optimal control | Time-dependent | | | | 73 | 6258 | 1209.57 |

Table S3 Results under C1–C5 and quadruple optimal control
The quadruple constant control settings C1–C5 are used for comparison with quadruple optimal control measures. Total infections indicate the cumulative number of infections. Infections averted are calculated relative to the scenario without control, in which cumulative infections reach 6331. Control-only cost represents the accumulated cost of each control strategy.

**Control profiles for Scenario A-D (single, dual, triple, and quadruple control measures)**

Fig. S1 Optimal control for the mpox of scenario A

Fig. S2 Optimal control for the mpox of scenario B

Fig. S3 Optimal control for the mpox of scenario C

Fig. S4 Optimal control for the mpox of scenario D
