# Supplementary material for: Dynamic modeling and optimal control of Mpox among MSM in a central Chinese city
Source: BMC Public Health. 2026 Aug 15;26:3351. doi: 10.1186/s12889-026-28925-6 (PMC13753281; doi:10.1186/s12889-026-28925-6)
Supplement: Supplementary file 2 [file 12889_2026_28925_MOESM2_ESM.docx]

**Additional file 2**

**Dynamic modeling and optimal control of Mpox among MSM in a central Chinese city**

Dan Wang, Si Wu, Siyu Zhang, Han Ni, Yuxi He, Liuyi Fu, Jin Zhao, Yuan Lv, Wenting Zha

**Contents**

[1. Sensitivity analysis of transmission dynamics model parameters	1](#_Toc18176)

[2. Sensitivity analysis of control measures	3](#_Toc17201)

*[2.1 MSM community based prevention and control (u](#_Toc27607)_[1](#_Toc27607)_[)](#_Toc27607)*	[3](#_Toc27607)

*[2.2 Vaccination (u](#_Toc18602)*_[4](#_Toc18602)_*[)](#_Toc18602)*	[6](#_Toc18602)

**1. Sensitivity analysis of transmission dynamics model parameters**

To assess the robustness of the optimal control conclusions to uncertainty in the transmission dynamics model parameters, we performed a Latin hypercube sampling (LHS) analysis for six epidemiological parameters in the model. These parameters included the baseline transmission rate for symptomatic infections (*β*), the proportion of asymptomatic infections (*p*), the relative infectiousness of asymptomatic infections (*η*_A_), the proportion of diagnosed symptomatic cases ($\varphi$), the removal rate of diagnosed symptomatic individuals (*γ*), and the recovery rate of asymptomatic and undiagnosed symptomatic individuals (*δ*). The corresponding ranges were 0.117895–0.136034 (*β*), 0.1215–0.1485 (*p*), 0.45–0.55 (*η*_A_), 0.315–0.385 ($\varphi$), 0.135–0.165 (*γ*), and 0.045–0.055 (*δ*), with most parameters varied by approximately ±10% around their baseline values. We first generated 100 LHS parameter sets. Because the basic reproduction number *R*_0_ is a derived quantity jointly determined by these parameters, sampling in the original parameter space does not guarantee uniform coverage of the target *R*_0_ range. Therefore, we further included the baseline parameter set, parameter sets close to the lower and upper boundaries of *R*_0_, and additional stratified parameter sets in sparsely sampled *R*_0_ intervals. This procedure yielded 126 parameter sets covering an *R*_0_ range of approximately 1.5–2.3. For each parameter set, the cumulative number of infections without control measures was recalculated. Under the same parameter set, we then computed the number of infections averted, the control-only cost, and other summary indicators for all 15 optimal control schemes. The results across the 126 parameter sets were further summarized to assess whether the main conclusions regarding the optimal control schemes were robust to changes in the transmission dynamics model parameters.

Table S4 Robustness of optimal control conclusions under model parameter changes

| Conclusions | Count of TRUE | Percentage |
| --- | --- | --- |
| Quadruple control averted the largest number of infections among all 15 schemes | 126 | 100% |
| *u*_1_ averted the largest number of infections among single control measures | 126 | 100% |
| *u*_1_+*u*_4_ averted the largest number of infections among dual control measures | 117 | 92.86% |
| *u*_1_+*u*_3_+*u*_4_ averted the largest number of infections among triple control measures | 126 | 100% |

Fig. S5 Percentage of infections averted by the 15 optimal control schemes under model parameter changes

Table S4 summarizes the robustness of the main conclusions regarding the optimal control strategies to changes in the transmission dynamics model parameters. Across the 126 parameter sets, quadruple control measures *u*_1_+*u*_2_+*u*_3_+*u*_4_ avert the largest number of infections among all 15 schemes in all parameter sets. Among single control measures, *u*_1_ achieves the best performance and averts the largest number of infections in all 126 parameter sets. Among dual control measures, *u*_1_+*u*_4_ averts the largest number of infections in 117 of the 126 parameter sets (92.86%). Among triple control measures, *u*_1_+*u*_3_+*u*_4_ averts the largest number of infections in all 126 parameter sets. These results are consistent with the main analysis and indicate that the main conclusions regarding the optimal control strategies are generally robust to changes in the transmission dynamics model parameters.

Fig. S5 shows the percentage of infections averted by the 15 optimal control schemes across the *R*_0_ range represented by the 126 parameter sets. The y-axis is displayed on a non-uniform scale to better visualize differences among schemes with high percentages of infections averted. Overall, the percentage of infections averted tends to increase as *R*_0_ increases, although local fluctuations are observed because similar *R*_0_ values can be generated by different parameter sets. Schemes including MSM community based prevention and control (*u*_1_) generally remain in the upper range, and quadruple control measures consistently provide the most effective overall strategy. These patterns are consistent with Table S4 and further support the robustness of the main conclusions to changes in the transmission dynamics model parameters.

**2. Sensitivity analysis of control measures**

*2.1 MSM community based prevention and control (u_1_)*

To assess the robustness of the optimal control conclusions to uncertainty in the upper bound and cost weight of MSM community based prevention and control (*u*_1_), we performed a sensitivity analysis for *u*_1_. Four upper bounds of *u*_1_ were considered, namely *u*_1,max_=0.5, 0.6, 0.7, and 0.8, and three cost weights were considered, namely *C*_1_=40, 50, and 60. This yielded 12 settings for MSM community based prevention and control (*u*_1_). Other model parameters, the upper bounds of *u*_2_, *u*_3_, and *u*_4_, the cost weights of *u*_2_, *u*_3_, and *u*_4_, simulation duration, and the 15 optimal control schemes were kept the same as in the main analysis. For each setting, we recalculated the cumulative number of infections, infections averted, and control-only cost under no control measures and all 15 optimal control schemes, and then assessed whether the main conclusions regarding the optimal control schemes were robust to these changes.

Table S5 shows that, across the 12 settings, quadruple control measures consistently avert the largest number of infections among the 15 optimal control schemes. With a single control measure, MSM community based prevention and control (*u*_1_) achieves the best performance in all settings. For dual control measures, *u*_1_+*u*_4_ averts the largest number of infections. For triple control measures, *u*_1_+*u*_3_+*u*_4_ also achieves the strongest control effect. These results indicate that changing the upper bound and cost weight of *u*_1_ within the tested ranges does not change the main conclusions regarding the relative performance of the optimal control schemes.

Table S5 Sensitivity analysis of MSM community based prevention and control (*u*_1_) settings for optimal control schemes

| *u*_1_ upper bound | *C_1_* | Scheme averting the largest number of infections overall | Scheme averting the largest number of infections among single control measures | Scheme averting the largest number of infections among dual control measures | Scheme averting the largest number of infections among triple control measures |
| --- | --- | --- | --- | --- | --- |
| 0.5 | 40 | *u*_1_+*u*_2_+*u*_3_+*u*_4_ | *u*_1_ | *u*_1_+*u*_4_ | *u*_1_+*u*_3_+*u*_4_ |
| 0.5 | 50 | *u*_1_+*u*_2_+*u*_3_+*u*_4_ | *u*_1_ | *u*_1_+*u*_4_ | *u*_1_+*u*_3_+*u*_4_ |
| 0.5 | 60 | *u*_1_+*u*_2_+*u*_3_+*u*_4_ | *u*_1_ | *u*_1_+*u*_4_ | *u*_1_+*u*_3_+*u*_4_ |
| 0.6 | 40 | *u*_1_+*u*_2_+*u*_3_+*u*_4_ | *u*_1_ | *u*_1_+*u*_4_ | *u*_1_+*u*_3_+*u*_4_ |
| 0.6 | 50 | *u*_1_+*u*_2_+*u*_3_+*u*_4_ | *u*_1_ | *u*_1_+*u*_4_ | *u*_1_+*u*_3_+*u*_4_ |
| 0.6 | 60 | *u*_1_+*u*_2_+*u*_3_+*u*_4_ | *u*_1_ | *u*_1_+*u*_4_ | *u*_1_+*u*_3_+*u*_4_ |
| 0.7 | 40 | *u*_1_+*u*_2_+*u*_3_+*u*_4_ | *u*_1_ | *u*_1_+*u*_4_ | *u*_1_+*u*_3_+*u*_4_ |
| 0.7 | 50 | *u*_1_+*u*_2_+*u*_3_+*u*_4_ | *u*_1_ | *u*_1_+*u*_4_ | *u*_1_+*u*_3_+*u*_4_ |
| 0.7 | 60 | *u*_1_+*u*_2_+*u*_3_+*u*_4_ | *u*_1_ | *u*_1_+*u*_4_ | *u*_1_+*u*_3_+*u*_4_ |
| 0.8 | 40 | *u*_1_+*u*_2_+*u*_3_+*u*_4_ | *u*_1_ | *u*_1_+*u*_4_ | *u*_1_+*u*_3_+*u*_4_ |
| 0.8 | 50 | *u*_1_+*u*_2_+*u*_3_+*u*_4_ | *u*_1_ | *u*_1_+*u*_4_ | *u*_1_+*u*_3_+*u*_4_ |
| 0.8 | 60 | *u*_1_+*u*_2_+*u*_3_+*u*_4_ | *u*_1_ | *u*_1_+*u*_4_ | *u*_1_+*u*_3_+*u*_4_ |

*2.2 Vaccination (u*_4_*)*

To assess the robustness of the optimal control conclusions to uncertainty in the assumptions on Vaccination (*u*_4_), we performed a sensitivity analysis for vaccine effectiveness and delayed vaccine protection. In the main model, Vaccination (*u*_4_) acts on the susceptible population, and susceptible individuals are transferred into the removed compartment *R* at the effective vaccination transition rate *κu*_4_, where *κ*=0.02. This setting assumes that vaccination confers complete protection during the control period. In the sensitivity analysis, the vaccination term *κu*_4_(*t*)*S*(*t*) was replaced by *κu*_4_^eff^(*t*)*S*(*t*). Without delayed vaccine protection, *u*_4_^eff^(*t*) was defined as *ε*_v_*u*_4_(*t*). With delayed vaccine protection, *u*_4_^eff^(*t*) was set to 0 before *t*_s_+14, and was defined as *ε*_v_*u*_4_(*t*−14) thereafter, where *t*_s_ denotes the intervention start time. Four levels of vaccine effectiveness were considered, namely *ε*_v_=0.70, 0.80, 0.90, and 1.00, and each level was evaluated with and without a delay of 14 days. This yielded eight settings for Vaccination (*u*_4_). Other model parameters, upper bounds of control variables, cost weights, simulation duration, and the 15 optimal control schemes were kept the same as in the main analysis. For each setting, we recalculated the cumulative number of infections, infections averted, and control-only cost under no control measures and all 15 optimal control schemes, and then assessed whether the main conclusions regarding the optimal control schemes were robust to these changes.

Table S6 shows that, across the eight settings, quadruple control measures consistently avert the largest number of infections among the 15 optimal control schemes, indicating that reduced vaccine effectiveness or delayed vaccine protection does not change the main conclusion regarding the most effective overall strategy. Among single control measures, MSM community based prevention and control (*u*_1_) consistently averts the largest number of infections. The relative ranking of combinations including Vaccination (*u*_4_) changes across different settings. Among dual control measures, *u*_1_+*u*_4_ averts the largest number of infections when vaccine effectiveness is 0.90 or 1.00 without delayed vaccine protection, whereas *u*_1_+*u*_3_ ranks first under the other settings. Among triple control measures, *u*_1_+*u*_3_+*u*_4_ averts the largest number of infections in all settings without delayed vaccine protection and in the setting with delayed vaccine protection and vaccine effectiveness of 1.00, whereas *u*_1_+*u*_2_+*u*_3_ ranks first when delayed vaccine protection is combined with lower vaccine effectiveness. Overall, assumptions related to vaccination mainly affect the ranking of schemes including *u*_4_, but do not change the conclusions that *u*_1_ is the best single control measure and that quadruple control measures provide the most effective overall strategy.

Table S6 Sensitivity analysis of Vaccination (*u*_4_) settings for optimal control schemes

| Vaccine effectiveness | Delay in vaccine protection | Scheme averting the largest number of infections overall | Scheme averting the largest number of infections among single control measures | Scheme averting the largest number of infections among dual control measures | Scheme averting the largest number of infections among triple control measures |
| --- | --- | --- | --- | --- | --- |
| 0.7 | 0 | *u*_1_+*u*_2_+*u*_3_+*u*_4_ | *u*_1_ | *u*_1_+*u*_3_ | *u*_1_+*u*_3_+*u*_4_ |
| 0.8 | 0 | *u*_1_+*u*_2_+*u*_3_+*u*_4_ | *u*_1_ | *u*_1_+*u*_3_ | *u*_1_+*u*_3_+*u*_4_ |
| 0.9 | 0 | *u*_1_+*u*_2_+*u*_3_+*u*_4_ | *u*_1_ | *u*_1_+*u*_4_ | *u*_1_+*u*_3_+*u*_4_ |
| 1 | 0 | *u*_1_+*u*_2_+*u*_3_+*u*_4_ | *u*_1_ | *u*_1_+*u*_4_ | *u*_1_+*u*_3_+*u*_4_ |
| 0.7 | 14 | *u*_1_+*u*_2_+*u*_3_+*u*_4_ | *u*_1_ | *u*_1_+*u*_3_ | *u*_1_+*u*_2_+*u*_3_ |
| 0.8 | 14 | *u*_1_+*u*_2_+*u*_3_+*u*_4_ | *u*_1_ | *u*_1_+*u*_3_ | *u*_1_+*u*_2_+*u*_3_ |
| 0.9 | 14 | *u*_1_+*u*_2_+*u*_3_+*u*_4_ | *u*_1_ | *u*_1_+*u*_3_ | *u*_1_+*u*_2_+*u*_3_ |
| 1 | 14 | *u*_1_+*u*_2_+*u*_3_+*u*_4_ | *u*_1_ | *u*_1_+*u*_3_ | *u*_1_+*u*_3_+*u*_4_ |
